# 中国医科大学成功举办“肺癌论坛-2018沈阳”高端学术大会并签署中美肺癌研究与临床诊治合作协议

**DOI:** 10.3779/j.issn.1009-3419.2018.08.13

**Published:** 2018-08-20

**Authors:** 军 张

**Affiliations:** 110001 沈阳，中国医科大学肺癌中心

**Keywords:** 肺癌论坛-2018沈阳, 中国医科大学肺癌中心, 国际肺癌研究学会, Lung Cancer Forum - 2018 Shenyang, China Medical University Lung Cancer Center (CLCC), International Association for the Study of Lung Cancer (IASLC)

由中国医科大学主办、中国医科大学肺癌中心承办的“肺癌论坛-2018沈阳”高端学术大会，于2018年7月21日-22日在沈阳成功举办。

中国科学技术协会副主席徐延豪教授莅临大会并做高屋建瓴的讲话，指出“2030健康战略”是习近平总书记非常关注、要求一定要解决好的民生问题，“没有健康就没有小康”是习总书记反复强调的战略问题。徐延豪副主席对大会主题“传播知识，增进友谊，齐心协力，战胜肺癌”和“以病人获益为中心，为肺癌病人谋福祉”，给予了充分肯定；尤其对成立“肺癌诊断与微创综合治疗专业委员会”、建立“辽宁省肺癌多中心多学科诊治平台”，并着手开展技术下基层、直接到辽宁省内各市县医院开展肺癌诊治技术提高班、真正惠及基层医院及中小城市与乡村肺癌病人等务实计划与行动表示赞同和支持，指明其对于真正实现国家分级诊疗大政方针具有重大现实意义，指示应注重积累经验，尤其是使其具有更大的可操作性、可复制性，利于推广，以惠及更多市县基层地区。

对中国医科大学肺癌中心与世界最大的肺癌研究与临床诊治专家组织——国际肺癌研究学会（International Association for the Study of Lung Cancer, IASLC）签署中美肺癌研究与临床诊治合作协议，以及联合沈阳市科协、由三方合作开展为期1个月的肺癌科普宣传月，徐延豪副主席均给予充分肯定和赞杨，并表示将号召北京、上海等全国更多城市共同参加这一肺癌防治的科普宣传活动。

徐延豪副主席对不远万里来到中国沈阳、帮助中国提高肺癌防治能力的世界肺癌大会主席、国际肺癌研究学会执行主任（首席执行官）、本次大会共同主席弗雷德•赫斯（Fred Hirsch）教授表示热烈欢迎和感谢。

**1:**
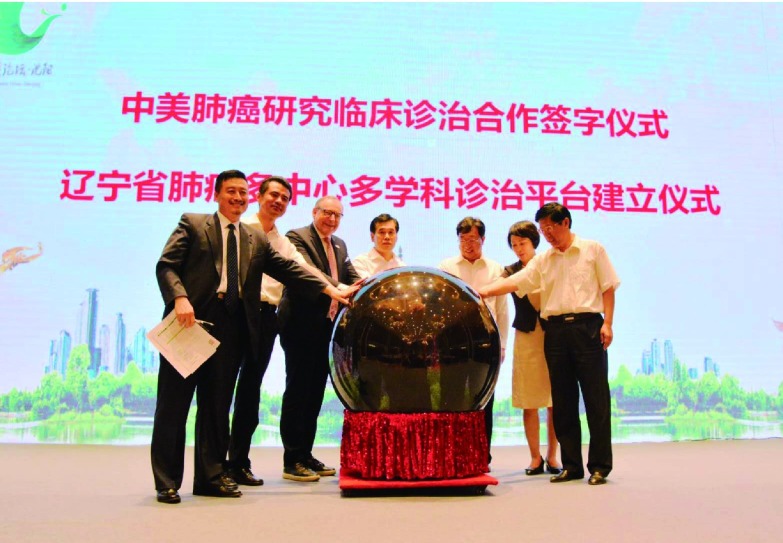
徐延豪、赫捷、弗雷德•赫斯、朱京海、张春英、吴智丰、张军等共同启动大会开幕。

**2:**
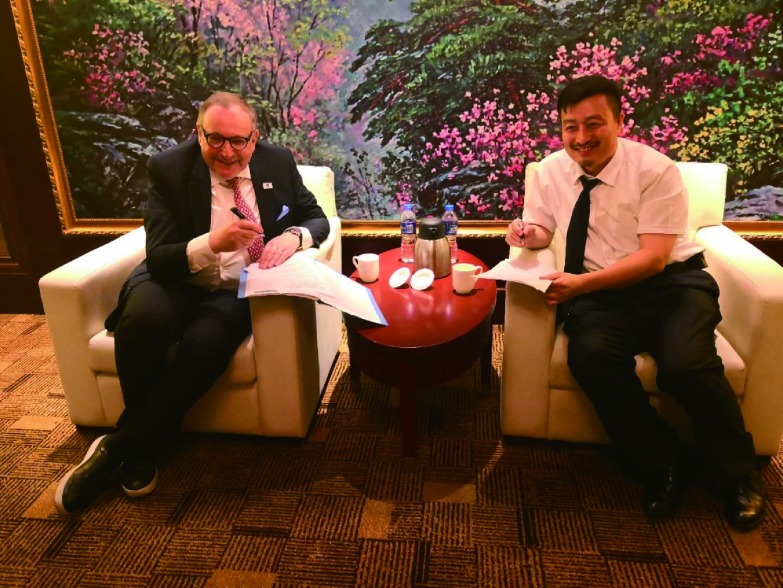
弗雷德•赫斯主席与张军教授共同签署、交换中美肺癌研究与临床诊治合作协议。

对中国科学院院士、国家癌症中心主任、中国医学科学院肿瘤医院院长、肺癌中心主任赫捷教授坐阵指挥、统领全国肺癌防治大略、将防治重点向全国更广大经济欠发达地区、基层地区扩展，给予了充分认可；对赫捷院士担任大会主席、身体力行推广学术、宣扬肺癌早诊早治、预防为主、全民共同努力的表帅精神和行动表示感谢。

对大会副主席——来自复旦大学附属肿瘤医院陈海泉院长、四川大学华西医院肺癌中心主任、天津医科大学原副校长周清华教授、安微医科大学附属第一医院副院长费广鹤教授、中日友好医院刘德若教授、复旦大学附属中山医院王群教授、山东省肿瘤医院邢力刚教授等国内肺癌外科、内科、放射治疗的顶级专家等以及辽宁省内各所医科大学附属医院、市县基层医院的教授、专家等，积极参与肺癌防治、科普宣传等表示感谢。

中国医科大学党委书记朱京海教授对徐延豪副主席莅临“肺癌论坛-2018沈阳”表示感谢，对大会主席赫捷院士、赫斯首席执行官对中国医科大学、中国医科大学肺癌中心的强有力支持表示热烈欢迎和诚挚感谢。对与会所有专家对中国医科大学的支持表示欢迎和感谢。

大会主席赫捷院土、弗雷德•赫斯首席执行官分别致大会主席欢迎词；作为国内、国际肺癌研究与临床诊治的“总舵主”，分别代表国内、国外肺癌研究与临床诊治的专家、学者以及学会组织，对“肺癌论坛-2018沈阳”的举办表示祝贺，并分别提供了各自的、具体可实施的支持计划。

大会执行主席、中国医科大学肺癌中心执行主任张军教授对莅临大会指导与讲座、分享与提高的科协领导徐延豪副主席、大会主席赫捷院土与弗雷德•赫斯首席执行官、国际及国内肺癌诊治各专业最著名专家、辽宁省内各市县基层医院肺癌诊治各科室主任及骨干力量，对《*Journal of Thoracic Oncology*》、《*Thoracic Cancer*》、《*Journal of Thoracic Disease*》、《中华肿瘤杂志》、《中国肺癌杂志》、《中国微创外科杂志》、《中华结核和呼吸杂志》等十余家SCI杂志及国内核心期刊的支持，表示热烈欢迎、诚挚感谢。对中国科协领导以及辽宁省科协党组书记张春英副主席、沈阳市科协党组书记吴智丰副主席等希望能够把大会办成每年在沈阳举办的具有国际水平的高端品牌会议表示感谢，并将尽心竭力办到、办好。对国际肺癌研究学会执行主任（首席执行官）给予新建学会“肺癌诊断与微创综合治疗专业委员会”的强大支持，包括签署合作协议以帮助派遣年轻医生赴美培训、开展肺癌临床诊治等全方位合作表示欢迎和感谢。张军教授号召全省肺癌诊治相关各专业医生积极参与、快速提高、为中国肺癌防治事业做出每一位医生本应有的更大的贡献。

在随后的大会论坛中，赫捷院士、弗雷德•赫斯主席以及陈海泉、周清华、费广鹤、刘德若、王群、邢力刚、韩晓红、李文慧、周宝森、张军、王琪、柳晨、韩玥、冯威建、吴楠、马锐、刘基巍、艾斌、耿庆妍教授等国内肺癌外科、内科、放射治疗等专业的顶级专家，辽宁省内各所医科大学附属医院、市县基层医院的教授、主任等分别进行大会报告，重点关注肺癌预防、早期筛查、早期发现，肺癌微创手术时机、切除范围与术后心肺功能等快速康复，肺癌二代测序技术（next-generation sequencing technology, NGS）检测与精准化疗、靶向治疗，肺癌精准放疗与免疫治疗，肺癌穿刺活检与微创综合治疗，以及医学论文的写作与提高、SCI杂志投稿策略等当今国内外肺癌热点、难点主题内容，并与参会代表进行了热烈而深入的讨论，报告专家与参会代表对相关肺癌研究与临床诊治的热点、难点问题唇枪舌剑、讨论热烈，报告专家与参会代表对相关话题均表现出浓厚的兴趣、进行了“很过瘾”的讨论、但由于每位报告专家的报告时间所限、双方都大有意犹未尽的感觉，相约一定要创造机会、再度“华山论剑”。大会报告、讨论，均非常成功。

大会共同主席、中国工程院院士、山东省肿瘤医院院长于金明教授，大会共同副主席四川大学华西医院院长李为民教授、广州医科大学附属第一医院院长何建行教授，因临时有重要任务而不能到会，分别向大会请假、专程致电向大会表示祝贺，并分别派专人参会、贺会。

大会决定，“肺癌论坛-2019沈阳”将于2019年7月19日-22日在沈阳举办，欢迎国内、国外肺癌专家、学者，大学附属医院医学教授、护理专家、青年医生、研究生、大学生，市县基层医院各级医生、护士，以及热心关怀肺癌事业的社会各界朋友，届时再度欢聚沈阳，“传播知识，增进友谊，齐心协力，战胜肺癌”，“以病人获益为中心，为肺癌病人谋福祉”。2019再相聚！

